# Serpentine bacteria influence metal translocation and bioconcentration of *Brassica juncea* and *Ricinus communis* grown in multi-metal polluted soils

**DOI:** 10.3389/fpls.2014.00757

**Published:** 2015-01-05

**Authors:** Ying Ma, Mani Rajkumar, Inês Rocha, Rui S. Oliveira, Helena Freitas

**Affiliations:** ^1^Centre for Functional Ecology, Department of Life Sciences, University of CoimbraCoimbra, Portugal; ^2^Department of Life Sciences, Central University of Tamil NaduThiruvarur, India; ^3^Centro de Biotecnologia e Química Fina – Laboratório Associado, Escola Superior de Biotecnologia, Universidade Católica PortuguesaPorto, Portugal; ^4^Research Centre on Health and Environment, School of Allied Health Sciences, Polytechnic Institute of PortoVila Nova de Gaia, Portugal

**Keywords:** phytostabilization, plant growth promoting bacteria, serpentine soils, heavy metals, *Brassica juncea*, *Ricinus communis*

## Abstract

The aim of this study was to assess the effects of inoculation of rhizosphere or endophytic bacteria (*Psychrobacter* sp. SRS8 and *Pseudomonas* sp. A3R3, respectively) isolated from a serpentine environment on the plant growth and the translocation and accumulation of Ni, Zn, and Fe by *Brassica juncea* and *Ricinus communis* on a multi-metal polluted serpentine soil (SS). Field collected SS was diluted to 0, 25, 50, and 75% with pristine soil in order to obtain a range of heavy metal concentrations and used in microcosm experiments. Regardless of inoculation with bacteria, the biomass of both plant species decreased with increase of the proportion of SS. Inoculation of plants with bacteria significantly increased the plant biomass and the heavy metal accumulation compared with non-inoculated control in the presence of different proportion of SS, which was attributed to the production of plant growth promoting and/or metal mobilizing metabolites by bacteria. However, SRS8 showed a maximum increase in the biomass of the test plants grown even in the treatment of 75% SS. In turn, A3R3 showed maximum effects on the accumulation of heavy metals in both plants. Regardless of inoculation of bacteria and proportion of SS, both plant species exhibited low values of bioconcentration factor (<1) for Ni and Fe. The inoculation of both bacterial strains significantly increased the translocation factor (TF) of Ni while decreasing the TF of Zn in both plant species. Besides this contrasting effect, the TFs of all metals were <1, indicating that all studied bacteria–plant combinations are suitable for phytostabilization. This study demonstrates that the bacterial isolates A3R3 and SRS8 improved the growth of *B. juncea* and *R. communis* in SS soils and have a great potential to be used as inoculants in phytostabilization scenarios of multi-metal contaminated soils.

## INTRODUCTION

Remediation of metal contaminated soils using plant based approaches (phytoremediation) is considered a simple, lower cost, environmentally friendly technology that can provide full-scale remediation when compared with existing physicochemical technologies (Ali et al., 2013). The most common phytoremediation method for soil remediation is phytostabilization, which utilizes metal tolerant plants to reduce/prevent the mobility of heavy metals in soils (Bolan et al., 2011). Currently, various plant species such as *Erica australis*, *E. andevalensis*, *Globularia alypum* L., *Rosmarinus officinalis* L., and *Solanum nigrum* L. have been used to reduce the mobility of heavy metals in artificially polluted soils (Ferraz et al., 2012; Testiati et al., 2013; Pérez-López et al., 2014). Although these plant species are able to grow on metal polluted soils, their application in phytostabilization practices in metal polluted fields is limited due to their low ability to adapt to adverse environmental conditions (Ma et al., 2011a). In general, multiple stresses including low soil pH, high salinity, low essential nutrients, and high concentrations of metals present in polluted field soils may limit the growth and survival of plants and thus compromise the overall phytoremediation process. Therefore, revegetation of metal polluted soils calls not only for the selection of appropriate plants species that are able to tolerate multiple stresses and accumulate/detoxify heavy metals but also the knowledge on the interactions among plant, soil, metals, and microbes.

In recent years, microbially enhanced methods of phytostabilization have been proposed as a viable strategy for removing/inactivating heavy metals in polluted soils (Grandlic et al., 2008). To reach such a goal of enhanced phytostabilization, the microbial inoculants must alleviate plant toxicity produced by heavy metals and increase plant tolerance. Metal resistant bacteria, particularly the rhizobacteria and endophytes, have been the most common plant associated beneficial microbes used as inoculants in phytostabilization processes of metal polluted soils (Rajkumar et al., 2013a; Jebara et al., 2014). These bacteria are capable of stimulating the plant growth and reducing metal uptake by producing various metabolites including 1-aminocyclopropane-1-carboxylic acid (ACC) deaminase, siderophores, and plant growth hormones, and mobilizing/immobilizing/transforming heavy metals (Zaidi et al., 2006; Dell’Amico et al., 2008; Rajkumar et al., 2012, 2013b; Becerra-Castro et al., 2013; Ma et al., 2013; Zhu et al., 2014). For example, He et al. (2013) have recently investigated the potential of inoculating *Brassica napus* with ACC utilizing bacteria *Rahnella* sp. JN6 to alleviate metal stress in plants and showed that plant height, root length, above-ground, and root weight were greatly increased. Similarly, Srivastava and Singh (2014) used the metal immobilizing plant growth promoting bacteria (PGPB) *Acinetobacter* sp. *nbri05* isolated from soil collected from an arsenic contaminated site to improve the plant growth and reduce the heavy metal translocation into plant shoots, thereby improving the phytostabilization potential of *Cicer aritenum* grown in arsenic contaminated soils. It has also been demonstrated that the use of such stress adapted microbial strains in phytostabilization studies is more effective than applying non-adapted strains (Rajkumar et al., 2013a; Jebara et al., 2014). Recently, Rajkumar et al. (2013a) investigated the phytostabilization potential of *Brassica juncea*, *Luffa cylindrica*, and *Sorghum halepense* after inoculation of Ni resistant serpentine isolate *Bacillus megaterium* SR28C and found that it was able to alleviate the detrimental effects of Ni by reducing its uptake and translocation to the plants.

Some progress has been made towards understanding the serpentine microbial diversity (Mengoni et al., 2004; Doherty et al., 2008) and their beneficial role on plant growth and phytoremediation in artificially Ni polluted soils (Ma et al., 2009a,b, 2010, 2011b; Rajkumar et al., 2013a), however, little is known about the role of serpentine bacterial isolates on the growth and heavy metal accumulation potential of plants in multi-metal polluted field soils. Heavy metals in general and Ni in particular present in SS were reported to have negative effect on plant mineral nutrition (Duman and Ozturk, 2010) and physiological metabolisms (Velikova et al., 2011) and henceforth the overall growth of plants. With the help of PGPB, the host plants can obtain more nutrients and plant resistance to heavy metal stress can be enhanced (Sessitsch et al., 2013).

In this study using SS as a model for multi-metal polluted field soils, we assessed the effects of inoculation of the metal resistant serpentine bacterial isolates *Pseudomonas* sp. A3R3 or *Psychrobacter* sp. SRS8 on the biomass production and Ni, Zn and Fe accumulation potential of *B. juncea* and *R. communis* plants. The aim was to evaluate the feasibility of using metal resistant-serpentine bacteria for microbe-assisted phytostabilization of contaminated soil.

## MATERIALS AND METHODS

### BACTERIAL STRAINS

The endophytic bacteria *Pseudomonas* spp. A3R3 (GenBank accession no. GU550663), and the rhizobacteria *Psychrobacter* sp. SRS8 (accession no. FM205059) originally isolated, respectively, from root interior and rhizosphere of *Alyssum serpyllifolium* grown in SS in Bragança, north–east of Portugal were obtained from the Culture Collection of the Center for Functional Ecology, University of Coimbra (Ma et al., 2009b, 2011b). The selection of these PGPB in this study was based on their ability to tolerate high concentrations of Ni and to produce plant growth promoting (PGP) substances (**Table 1**).

**Table 1:** Characteristic features of the bacterial strains used in this study.

	Plant growth promoting traits								
Bacterial strain	ACC deaminase (μm α-KB mg^-1^h^-1^)	P solubilization (mg L^-1^)	IAA production (mg L^-1^)	Siderophore production (mg L^-1^)		Ni resistance level (mg L^-1^)	Genbank accession no.	Source	Reference
				Catechol	Hydroxamate				
*Pseudomonas* sp. A3R3	67.9 ± 6.2	138 ± 21.4	69.4 ± 3.2	83.3 ± 7.5	60.5 ± 6.3	750	GU550663	Root of *Alyssum serpyllifolium*	Ma et al. (2011b)
*Psychrobacter* sp. SRS8	nd	126 ± 2.2	111 ± 1.4	899 ± 30.3	28.0 ± 2.2	1000	FM205059	Rhizosphere of *A. serpyllifolium*	Ma et al. (2009b)

*Average ± SD from three samples. nd, not detected; ACC, 1-aminocyclopropane-1-carboxylic acid; α-KB, α-ketobutyrate; IAA, indole-3-acetic acid.*

### EXPERIMENTAL PLANTS

Two crop plants, *B. juncea* (L.) Czern. and *Ricinus communis* L. were selected for this study based on their demonstrated ability to grow in heavily polluted soil (Bauddh and Singh, 2012) and to accumulate high amounts of heavy metals (Cecchi and Zanchi, 2005; Ma et al., 2009b) together with their potential to produce substantial biomass in a very short time (Safari Sinegani and Khalilikhah, 2008).

### MICROCOSM EXPERIMENTS

The SS used in the present study was collected from the Bragança, north-east of Portugal (Freitas et al., 2004), while the pristine soil (PS) was collected from the Botanical Garden of the University of Coimbra, Coimbra, Portugal. The physicochemical properties and heavy metals concentrations of the SS and PS are shown in ****Table 2****. Seeds of *B. juncea* and *R*. *communis* obtained from the Botanical Garden of the University of Coimbra, Portugal, were surface sterilized with 2% Ca(OCl)_2_ during 2 h and rinsed three times with autoclaved deionized water. The seeds were allowed to germinate in sterilized non-contaminated PS at 25°C under a 16/8 day/night regime. Seedlings were exposed to full sunlight during the day. The soil collected from the serpentine area was mixed with the PS at four different proportions: 0% SS (0% SS + 100% PS), 25% SS (25% SS + 75% PS), 50% SS (50% SS + 50% PS) and 75% SS (75% SS + 25% PS). The mixture was based on the dry weights of the soils. 100% SS (100% SS + 0% PS) was omitted in this study, since in our preliminary microcosm experiments both *B. juncea* and *R. communis* grown in 100% SS showed signs of wilting as early as 15 days after the transplantation, and they died during the experiments (data not shown). For inoculation of the seedlings, the bacterial strains were grown in Luria Bertani medium for 18 h at 27°C and 200 rpm. The cultures were then centrifuged at 7000 rpm for 10 min and the pellets were washed with biological saline (0.85% KCl). The pellet was re-suspended in biological saline and the colony forming unit (CFU) was adjusted to 10^9^ mL^-1^. Fifteen-day-old seedlings were inoculated by soaking the root system in the bacterial culture for 2 h. The roots of control plants were soaked in sterile deionized water. Seedlings were transplanted into plastic pots with a volume of 0.86 cm^3^, containing 300 g of 0, 25, 50, or 75% SS. The plants were allowed to grow in a greenhouse with an average temperature of 25°C and a 16:8 h day/night regime and watered as required. All treatments were performed in three replicates.

**Table 2:** Physicochemical properties of the soils used in this study.

Parameters	Serpentine soil		Pristine soil	
pH	7.5 - 8.5		7.3 – 7.5	
Organic matter (%)	1.3 ± 0.1		1.6 ± 0.2	
Cation exchange capacity [meq(100g)^-1^]	1.0 ± 0.2		1.5 ± 0.1	
Electrical conductivity (dS m^-1^)	0.8 ± 0.1		1.2 ± 0.2	

**Metals (mg kg^-**1**^)**	**Total**	**Extractable**	**Total**	**Extractable**

Ni	812 ± 35	4.3 ± 0.8	18 ± 1	nd
Zn	160 ± 12.3	48.7 ± 2.8	86 ± 10.5	9.3 ± 1.1
Fe	6849 ± 215	437 ± 12.1	653 ± 33	75.6 ± 12.1

*Average ± SD from three samples. nd, not detected.*

### SOIL ANALYSIS

Both total and diethylenetriaminepentaacetic acid (DTPA)-extractable fractions of heavy metals (Ni, Zn, and Fe) were measured in the soil. To analyze the total metal content, three replicates of 1 g of soil were weighed and 10.5 mL of *aqua regia* were added to each sample according to Kilburn (2000). The digestion tubes were allowed to stand overnight to equilibrate the *aqua regia* with the soil and then were put onto a hotplate for 3 h at 110°C. The digests were filtered through Whatman No. 42 filter paper and mixed with deionized water in volumetric flask reaching 25 mL. The concentration of heavy metals in soil extracts was measured by atomic absorption spectrophotometry (AAS; PerkinElmer model 100, MA, USA).

Diethylenetriaminepentaacetic acid-extractable metals were measured by extracting 10 g samples of soil with 20 mL of extracting solution (0.005 M DTPA, 0.01 M CaCl_2_, 0.1 M Triethanolamine, pH 7.3) according to the procedure of Lindsay and Norvell (1978). The mixtures were agitated on a horizontal shaker for 2 h at room temperature and were then through Whatman No. 42 filter paper prior to analysis by AAS.

### PLANT ANALYSIS

After 60 days, plants were harvested and the roots were carefully washed with deionized water and then with 10 mM ethylenediaminetetraacetic acid to remove all attached soil particles. The growth parameters such as root and shoot weight (fresh and dry) were determined. To evaluate the heavy metal concentrations, the washed plant materials were oven dried at 70°C for 3 days and finely ground to a powder. The metal contents (Ni, Zn, and Fe) in the plant tissues were measured by AAS after digestion in a mixture of concentrated HNO_3_ and HClO_4_ (4:1, v/v; Ma et al., 2009a).

The determinations of the metal concentration in plant tissues and soils were used to estimate the translocation factor (TF) and the bioconcentration factor (BCF). The TF was calculated by dividing shoot metal concentration by the root metal concentration ([Metal]*_shoot_*/[Metal]*_root_*; Lotfy and Mostafa, 2014) and the BCF was calculated by dividing the metal concentration in the entire plant by the initial soil metal concentration ([Metal]*_plant_/*[Metal]*_soil_*; Zayed et al., 1998). Metal concentrations in all compartments were calculated on a dry weight basis.

### STATISTICAL ANALYSIS

Data were analyzed by analysis of variance (ANOVA) and treatment means were compared using Fisher’s protected LSD test (*p* < 0.05). All the analyzes were performed using SPSS 10.0.

## RESULTS AND DISCUSSION

### PLANT GROWTH

**FIGURE 1:**
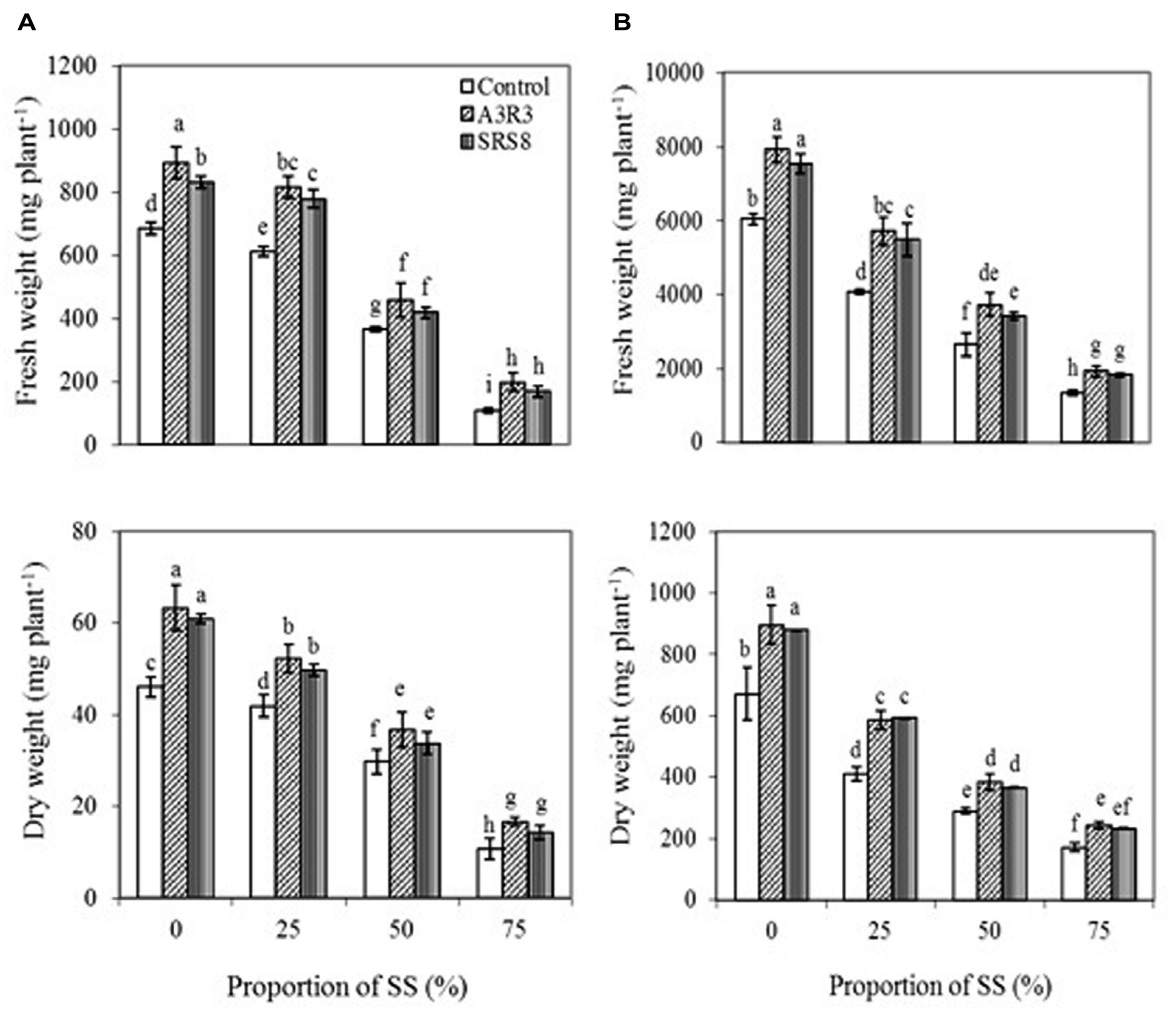
**Influence of *Psychrobacter* sp. SRS8 and *Pseudomonas* sp. A3R3 on the fresh weight and dry weight of *Brassica juncea* (A) and *Ricinus communis* (B) grown in different proportion of serpentine soil (SS)**. Each value is the mean of triplicates. Error bars represent SD. Data of columns indexed by the same letter are not significantly different according to Fisher’s protected LSD test (*p* < 0.05).

Although *B. juncea* and *R*. *communis* used in this study have been reported to grow in heavy metal contaminated soils (Niu et al., 2007; Bauddh and Singh, 2012), the adverse environmental conditions particularly the low essential nutrients and the elevated levels of various metals in SS (Freitas et al., 2004) may reduce the plant growth and biomass production through impairing their metabolism and interfering with the absorption of essential nutrients (Duman and Ozturk, 2010). Soil properties greatly influence plant growth and root development. The low organic matter (1.3–1.6%) and cation exchange capacity [1.0–1.5 meq(100g)^-1^] (**Table 2**) of the soils used in our study may have contributed to the limited growth of the tested plants. Thus in this study, the effect of rhizosphere or endophytic bacteria previously isolated from serpentine environment (Ma et al., 2009b, 2011b) on the growth and metal accumulation potential of *B. juncea* and *R*. *communis* in SS was investigated with the objective of using these species for phytoremediation of multi-metal polluted field soils. In control soils (0% SS), inoculation of *B. juncea* and *R*. *communis* with *Pseudomonas* sp. A3R3 or *Psychrobacter* sp. SRS8 showed an increase in plant fresh and dry weight (**Figures 1A,B**). However, maximum PGP effect was observed in A3R3. In the case of *B. juncea*, inoculation of A3R3 increased plant fresh and dry weight by 30 and 38%, respectively. Similarly, in *R*. *communis*, the strain A3R3 enhanced the fresh and dry weight by 31 and 34%, respectively. Recent studies on the mode of action of PGPB have shown that the increase in plant growth is due to microbial production of various PGP metabolites such as ACC deaminase (which can reduce stress ethylene production in plants through hydrolyzing ethylene precursor ACC to ammonia and α-ketobutyrate, α-KB; Arshad et al., 2007); indole-3-acetic acid (IAA) (which can enhance the plant growth by stimulating plant cell elongation or affecting cell division; Rashotte et al., 2000); nutrient solubilizing metabolites (which can mobilize nutrients – e.g., P and Fe in the rhizosphere; Sessitsch et al., 2013). The maximum plant growth promotion by A3R3 observed in the present study might have been due to the cumulative effects of the production of siderophores, ACC deaminase, IAA and solubilization of P (**Table 1**).

The non-inoculated plants exposed to different levels of SS (25–75%), showed a marked inhibition in the growth. The fresh and dry weight of the *B. juncea* and *R. communis* generally decreased with increasing the concentrations of SS. In the treatment with 75% SS, the growth of *B. juncea* was considerably decreased (**Figure 1A**), with an 84% reduction in fresh weight and 77% reduction in dry weight. Similar responses were also observed in *R. communis* (**Figure 1B**), where fresh and dry weight decreased by 78 and 75%, respectively. The low growth observed for plants in SS had already been reported by other authors (Abou-Shanab et al., 2006; Kayama et al., 2006). Plants inoculated with *Pseudomonas* sp. A3R3 or *Psychrobacter* sp. SRS8 exhibited an increase in plant growth in the presence of different levels of SS. However, inoculation with *Pseudomonas* sp. A3R3, a strain that showed ACC deaminase activity in addition to other PGP traits, resulted in higher fresh and dry weight of plants. *Pseudomonas* sp. A3R3 increased the fresh and dry weight of *B. juncea* even in the treatment of 75% SS by 82 and 55%, respectively, compared to non-inoculated plants grown with the same treatment of SS. Similarly, in *R. communis*, *Pseudomonas* sp. A3R3 enhanced the fresh and dry weight by 45 and 42%, respectively at 75% SS. Similar results have been previously reported by Ma et al. (2011b), Zhang et al. (2011), and Srivastava et al. (2013), suggesting that the production of ACC deaminase by strain A3R3, which can hydrolyze ethylene precursor ACC to α-KB and ammonia, may alleviate the Ni stress-mediated impact on plants. Further, *Pseudomonas* sp. A3R3 can also produce catechol and hydroxamate type siderophores, IAA and solubilize P (Ma et al., 2011b), indicating that both *B. juncea* and *R. communis* might have benefited from this strain. Since *Pseudomonas* sp. A3R3 was originally isolated from the root tissues of *A*. *serpyllifolium* (Ma et al., 2011b), the increased plant growth caused by *Pseudomonas* sp. A3R3 also implies that the endophytic bacteria with various PGP traits might be preferable for plant growth promotion in metal polluted soils. However, this has to be tested with a broader spectrum of endophytes before a general validity can be assumed since the present study comprised only one endophytic bacterial strain. Based on the above observations, it was inferred that the inoculation of serpentine PGPB improved the establishment of *B. juncea* and *R. communis* in Ni rich SS, as reflected by increased growth performance.

### HEAVY METAL CONCENTRATIONS IN SOILS AFTER HARVESTING

Influence of plants and PGPB inoculation on the variation of metal mobility in soils was determined after harvesting. Regardless of inoculation with PGPB, a decrease in the total content of all the three metals was observed in root-adhering soils of both *B. juncea* and *R. communis*, compared to unplanted (**Table 3**), as a likely consequence of metal uptake by plants. However, plants inoculated with PGPB were effective at decreasing the total Ni and Zn concentrations in soils compared with respective non-inoculated control. In the treatment with 75% of SS, inoculation of *Psychrobacter* sp. SRS8 decreased the total Ni and Zn concentrations in the soils of *B. juncea* by 28 and 25%, respectively, whereas in the soils of *R. communis* the percentage of decrease was 23 and 22, respectively. This result suggests that with the help of PGPB both *B. juncea* and *R. communis* can efficiently take up Ni and Zn from soils and confirm the previous finding that rhizosphere microbes may stimulate heavy metal uptake by plants (Chen et al., 2013; Prapagdee et al., 2013). On the contrary to total Ni and Zn contents, plants inoculated with PGPB significantly increased the concentration of total Fe in soils when compared with the non-inoculated control plants. This may be attributed to a decrease in plant Fe uptake, possibly due to the presence of high concentrations of bioavailable Ni or competition between bioavailable Ni and Fe at the plant uptake site as a consequence of microbial induced Ni mobilization. Previously, plant nutritional imbalances in response to the presence of heavy metals have been reported by Ouzounidou et al. (2006), suggesting that Ni may compete effectively for specific binding sites or by other means block the uptake units, leading to decrease in Fe uptake by plants.

**Table 3:** Heavy metals concentrations in soils before planting and after harvesting of *Brassica juncea* and *Ricinus communis* inoculated with *Psychrobacter* sp. SRS8 and *Pseudomonas* sp. A3R3.

Treatment	DTPA extractable (mg kg^-1^ dry soil)			Total (mg kg^-1^ dry soil)		
	Ni	Zn	Fe	Ni	Zn	Fe
**Before planting**
SS 0% (Pristine soil)	nd	1.0 ± 0.1	35.1 ± 6.4	21.6 ± 2.7	38.5 ± 6.8	556 ± 24.6
SS 25% (without plants )	nd	18.7 ± 0.9	159 ± 10.7	290 ± 46.2	180 ± 25.2	2290 ± 217
SS 50% (without plants)	8.9 ± 3.3	28.7 ± 6.8	220 ± 54.3	367 ± 58.9	214 ± 56.4	3664 ± 190
SS 75% (without plants)	14.8 ± 3.3	33.9 ± 9.7	190 ± 23.6	418 ± 89.6	235 ± 17.5	5311 ± 218
**After harvesting of *B. juncea***
SS 0%	nd	2.2 ± 0.7 g	54.0 ± 10.5 h	19.2 ± 1.7 f	34.4 ± 5.7 e	395 ± 28.4 i
SS 0% + A3R3	nd	18.6 ± 4.4 f	73.8 ± 8.4 g	14.5 ± 0.9 f	29.4 ± 2.9 e	468 ± 68.0 hi
SS 0% + SRS8	nd	62.3 ± 9.4 c	83.3 ± 12.6 g	12.6 ± 1.2 f	22.7 ± 7.1 e	502 ± 39.0 h
SS 25%	1.6 ± 0.4 h	21.3 ± 1.5 f	202 ± 20.6 f	252 ± 37.8 d	167 ± 34.7 c	1992 ± 367 f
SS 25% + A3R3	15.5 ± 2.6 f	51.2 ± 6.4 d	289 ± 17.3 c	189 ± 15.3 e	126 ± 13.0 d	1923 ± 194 fg
SS 25% + SRS8	28.0 ± 5.3 e	76.3 ± 7.9 b	338 ± 38.5 b	171 ± 13.5 e	107 ± 14.1 d	2070 ± 275 f
SS 50%	10.4 ± 2.6 g	35.4 ± 5.2 e	281 ± 31.6 cd	323 ± 19.3 b	196 ± 47.2 b	2775 ± 298 e
SS 50% + A3R3	43.8 ± 10.0 d	58.4 ± 9.1 c	378 ± 23.5 a	271 ± 12.5 c	153 ± 18.4 c	2998 ± 306 d
SS 50% + SRS8	74.2 ± 10.5 b	85.5 ± 7.3 a	384 ± 65.3 a	243 ± 8.2 d	127 ± 8.3 d	3284 ± 271 c
SS 75%	16.8 ± 2.9 f	38.0 ± 8.0 e	228 ± 26.1 e	364 ± 42.2 a	223 ± 29.1 a	4335 ± 407 b
SS 75% + A3R3	51.2 ± 16.2 c	51.8 ± 8.8 d	267 ± 37.2 d	309 ± 13.0 b	190 ± 10.3 b	4539 ± 400 a
SS 75% + SRS8	86.5 ± 7.3 a	73.0 ± 7.2 b	272 ± 35.0 cd	261 ± 15.8 cd	168 ± 13.3 bc	4416 ± 278 b
**After harvesting of *R. communis***
SS 0%	nd	16.5 ± 1.7 f	26.6 ± 5.2 g	26.8 ± 4.3 h	18.6 ± 3.5 e	290 ± 42.8 j
SS 0% + A3R3	nd	35.3 ± 3.3 e	43.0 ± 10.0 fg	20.6 ± 1.6 h	15.4 ± 3.2 e	335 ± 31.5 ij
SS 0% + SRS8	nd	50.9 ± 2.6 cd	58.6 ± 5.6 f	19.1 ± 1.9 h	11.8 ± 1.8 e	421 ± 31.6 i
SS 25%	0.9 ± 0.2 g	33.5 ± 3.2 e	112 ± 12.0 e	375 ± 20.9 d	102 ± 18.1 c	1242 ± 90.3 h
SS 25% + A3R3	7.5 ± 0.7 f	81.8 ± 5.7 b	218 ± 26.2 d	305 ± 37.8 f	79.6 ± 12.4 d	1387 ± 155 g
SS 25% + SRS8	13.9 ± 1.5 e	97.6 ± 7.3 a	218 ± 24.4 d	247 ± 31.0 g	78.4 ± 6.0 d	1542 ± 71.9 f
SS 50%	8.9 ± 1.4 f	56.5 ± 4.4 c	217 ± 18.0 d	449 ± 29.3 b	125 ± 32.6 b	2185 ± 244 e
SS 50% + A3R3	34.3 ± 4.5 d	79.2 ± 3.5 b	298 ± 47.0 b	350 ± 38.4 e	103 ± 20.9 c	2404 ± 265 d
SS 50% + SRS8	56.5 ± 3.9 b	96.1 ± 7.4 a	337 ± 24.8 a	326 ± 20.4 f	99.9 ± 12.5 c	2589 ± 225 c
SS 75%	15.8 ± 1.5 e	62.7 ± 3.8 c	218 ± 27.0 d	477 ± 31.4 a	151 ± 18.0 a	3644 ± 276 b
SS 75% + A3R3	37.7 ± 4.7 c	85.5 ± 6.4 b	219 ± 53.4 d	402 ± 22.9 c	119 ± 17.7 b	3836 ± 199 a
SS 75% + SRS8	67.8 ± 3.1 a	100 ± 11.4 a	246 ± 14.4 c	365 ± 45.5 de	119 ± 8.9 b	3707 ± 146 b

*Average ± SD from three samples. Data of columns within each plant species indexed by the same letter are not significantly different according to Fisher’s protected LSD test (*p* < 0.05). nd, not detected; DTPA, diethylenetriaminepentaacetic acid; SS, serpentine soil.*

According to Jackson and Alloway (1991), Singh et al. (1996), Rauret et al. (1999), Wang et al. (2004), and Meers et al. (2007), the levels of metals in plant tissues are closely related to the concentrations of DTPA-extractable metals in soils. Therefore, the bioavailable fraction of metals in the rhizosphere soils of *B. juncea* and *R communis* was determined using DTPA as extractant. In general, plant roots and rhizosphere microbes strongly influence the physicochemical characteristics of the soil through various metabolic actions/activities [e.g., excretion of organic acids, (phyto)siderophores] resulting in an increase/decrease in metal bioavailability in soils. In the present study, irrespective of inoculation of PGPB, the bioavailable fractions in the soils of all SS treatments increased significantly after the growth of *B. juncea* or *R communis* and this was likely due to the ability of plant and/or its associated microbes to increase metal availability (**Table 3**). However, this effect was higher when the plants were inoculated with PGPB, particularly *Psychrobacter* sp. SRS8. These results clearly revealed that inoculation of PGPB prompted the release of unavailable metals into soil. In the treatment of 50% SS, inoculation of *B. juncea* with *Psychrobacter* sp. SRS8 increased maximum concentrations of DTPA extractable Ni, Zn, and Fe in the soils, which were 7.1-, 2.4-, and 1.4-fold higher than those in the soils of non-inoculated plants, respectively. Similarly, *R communis* inoculated with *Psychrobacter* sp. SRS8 increased the concentrations of DTPA extractable Ni, Zn, and Fe in the soil by 6.3-, 1.7-, and 1.6-fold, respectively. The increase in DTPA extractable Ni, Zn, and Fe after bacterial inoculation might be attributed to solubilization of unavailable forms of heavy metal bearing minerals due to complexation reaction as a consequence of metabolites (e.g., organic acids, siderophores) release by PGPB.

### METAL UPTAKE BY *Brassica juncea* AND *Ricinus communis*

Metal concentrations in roots and shoots of both plants tended to increase in the treatments with higher proportions of SS (**Table 4**). The maximum accumulation of metals was observed in the root and shoot systems of plants grown in 75% SS. However, the metal concentrations in the shoots of both plants were much lower than in the respective roots. A similar finding was recently reported for *B. juncea* and *R. communis* grown on metal contaminated soils (Bauddh and Singh, 2012). Regardless of inoculation of PGPB, *B. juncea* took up more heavy metals than *R. communis* under the same conditions, except Zn which concentration was higher in root and shoot tissues of *R. communis*. Besides, the concentrations of Fe and Zn in the tissues of both plants were found to be higher than Ni and this could be explained by the fact that Zn and Fe are an essential element for plant growth (Desideri et al., 2010).

**Table 4:** Heavy metals concentrations in shoots and roots of *Brassica juncea* and *Ricinus communis* grown in serpentine soils (SS) and inoculated with *Psychrobacter* spp. SRS8 and *Pseudomonas* spp. A3R3.

Treatment		Shoot (mg kg^-1^ dw)			Root (mg kg^-1^ dw)		
Proportion of SS (%)	PGPB	Ni	Zn	Fe	Ni	Zn	Fe
	strain						
***B. juncea***
0	Control	2.2 ± 0.2 i	9.5 ± 0.6 h	67.0 ± 5.3 f	13.2 ± 1.3 e	17.8 ± 2.7 e	170 ± 23.3 f
	A3R3	4.4 ± 0.5 hi	10.1 ± 0.8 h	75.3 ± 4.5 f	14.9 ± 1.5 e	24.8 ± 4.5 e	196 ± 22.2 f
	SRS8	5.3 ± 0.3 h	11.1 ± 1.0 h	75.7 ± 7.3 f	17.2 ± 2.0 e	26.0 ± 3.9 e	215 ± 34.8 f
25	Control	29.7 ± 1.3 g	37.6 ± 1.9 g	86.0 ± 9.3 e	127 ± 6.5 d	138 ± 17.9 d	352 ± 32.4 e
	A3R3	34.0 ± 1.9 f	41.9 ± 4.6 f	109 ± 15.5 d	140 ± 2.1 c	176 ± 16.8 c	461 ± 51.8 d
	SRS8	39.7 ± 2.1 e	44.3 ± 5.1 f	117 ± 12.5 d	147 ± 8.3 c	189 ± 11.3 c	525 ± 37.3 d
50	Control	36.6 ± 2.0 ef	58.3 ± 3.4 e	115 ± 5.3 d	146 ± 6.5 c	177 ± 23.4 c	510 ± 42.9 d
	A3R3	39.7 ± 2.8 e	61.7 ± 5.2 de	135 ± 15.2 c	163 ± 14.3 b	219 ± 21.8 bc	640 ± 46.1 bc
	SRS8	42.8 ± 4.2 d	64.0 ± 5.5 d	198 ± 11.0 b	167 ± 6.5 b	232 ± 26.8 bc	667 ± 43.5 bc
75	Control	46.7 ± 1.7 c	72.3 ± 6.9 c	188 ± 17.8 b	173 ± 8.3 b	261 ± 12.7 b	615 ± 44.0 c
	A3R3	50.0 ± 1.9 b	83.3 ± 8.0 b	234 ± 15.2 a	187 ± 15.0 ab	287 ± 35.2 ab	706 ± 6.4 ab
	SRS8	54.4 ± 4.1 a	90.6 ± 5.1 a	244 ± 25.0 a	193 ± 13.1 a	304 ± 35.2 a	733 ± 86.8 a
***R. communis***
0	Control	1.7 ± 0.4 e	11.5 ± 0.8 f	26.4 ± 4.2 e	9.5 ± 0.7 f	28.5 ± 1.6 f	95.3 ± 3.1 g
	A3R3	3.0 ± 0.9 e	13.1 ± 1.0 f	34.1 ± 3.8 e	12.7 ± 0.8 f	40.0 ± 3.2 f	116 ± 8.6 g
	SRS8	3.3 ± 0.7 e	14.5 ± 0.9 f	33.5 ± 4.2 e	14.2 ± 0.8 f	44.3 ± 5.4 f	105 ± 9.2 g
25	Control	13.2 ± 1.2 d	23.9 ± 3.3 e	46.9 ± 3.9 d	90.4 ± 6.9 e	184.5 ± 8.2 e	157 ± 11.5 f
	A3R3	19.8 ± 3.4 c	34.3 ± 4.1 d	56.3 ± 5.9 c	109 ± 8.1 d	220 ± 24.8 d	187 ± 7.2 e
	SRS8	21.5 ± 3.4 c	35.9 ± 3.0 d	57.9 ± 5.0 c	112 ± 14.2 d	234 ± 5.4 d	205 ± 10.9 de
50	Control	22.1 ± 4.0 c	41.3 ± 4.9 cd	58.9 ± 7.9 c	113 ± 12.3 cd	219 ± 12.5 d	224 ± 10.1 d
	A3R3	29.2 ± 1.7 b	51.1 ± 4.0 b	70.0 ± 8.6 b	127 ± 4.9 bc	283 ± 16.6 c	265 ± 22.4 c
	SRS8	30.1 ± 2.9 b	53.2 ± 3.8 b	72.5 ± 6.6 b	129 ± 11.4 b	329 ± 18.5 b	273 ± 13.7 c
75	Control	31.4 ± 4.3 b	52.4 ± 4.7 b	75.0 ± 5.8 b	133 ± 18.2 b	287 ± 22.1 c	316 ± 21.9 b
	A3R3	38.9 ± 3.7 a	60.4 ± 8.4 a	92.0 ± 6.0 a	147 ± 9.4 ab	344 ± 16.0 ab	379 ± 20.3 a
	SRS8	40.5 ± 15.2 a	61.7 ± 8.2 a	93.9 ± 10.8 a	159 ± 9.6 a	358 ± 13.7 a	393 ± 12.0 a

*Average ± SD from three samples. Data of columns within each plant species indexed by the same letter are not significantly different according to Fisher’s protected LSD test (*p* < 0.05). dw, dry weight; PGPB, plant growth promoting bacteria.*

Inoculation of plants with PGPB greatly enhanced the quantity of accumulation of Ni in root and shoot tissues compared with respective non-inoculated controls (**Table 4**). However, the maximum effect was observed in *Psychrobacter* sp. SRS8. In the treatment with 75% of SS, *Psychrobacter* sp. SRS8 increased the Ni concentrations in the shoot and root tissues of *B. juncea* by 16 and 11%, respectively, whereas in *R. communis* the percentage of increase was 29 and 20, respectively. The increased Ni accumulation in both plants by *Psychrobacter* sp. SRS8 might be due to its efficiency of mobilizing the Ni at the soil-root interface. Our result is in agreement with the observations of Chen et al. (2013) and Prapagdee et al. (2013) who reported enhanced uptake of metals in *B. napus* and *H. annuus* due to inoculation of metal mobilizing PGPB suggesting that the inoculation of *Psychrobacter* sp. SRS8 enhances the heavy metal availability in the rhizosphere (Ma et al., 2009b) and thereby increases the uptake of Ni by plants (**Table 4**).

Zn and Fe concentrations in the roots of both plants inoculated with or without PGPB also showed similar trends to those described for Ni concentrations (**Table 4**). This finding is in accordance with other observation reported in Ma et al. (2010) and Babu et al. (2013), where Zn and Fe concentrations in the plant roots were greater than those in the shoots. The non-inoculated plants grown with various SS treatments showed low Zn and Fe concentrations, but PGPB inoculation increased Zn and Fe concentrations in the shoots and roots of both plant species grown under the same condition (**Table 4**). This may be attributed to the microbial metabolites/actions such as production of siderophores and P solubilization. Although both *Psychrobacter* sp. SRS8 and *Pseudomonas* sp. A3R3 showed similar trends on the production of siderophores and solubilization of P (**Table 2**), the rhizosphere isolate *Psychrobacter* sp. SRS8 was more effective than the endophytic strain A3R3 in stimulating heavy metal accumulation in the tissues of both plants. Most likely, *Psychrobacter* sp. SRS8 may have produced metal mobilizing metabolites in the rhizosphere and thus may have increased heavy metal availability to plant roots as they are usually in close contact with the root surface and heavy metals in the soils, and improved its uptake. Further work is, however, required to characterize the microbial metabolites and to elucidate the factors that induce heavy metal accumulation in plants.

### TRANSLOCATION OF METALS FROM ROOTS TO SHOOTS

**FIGURE 2:**
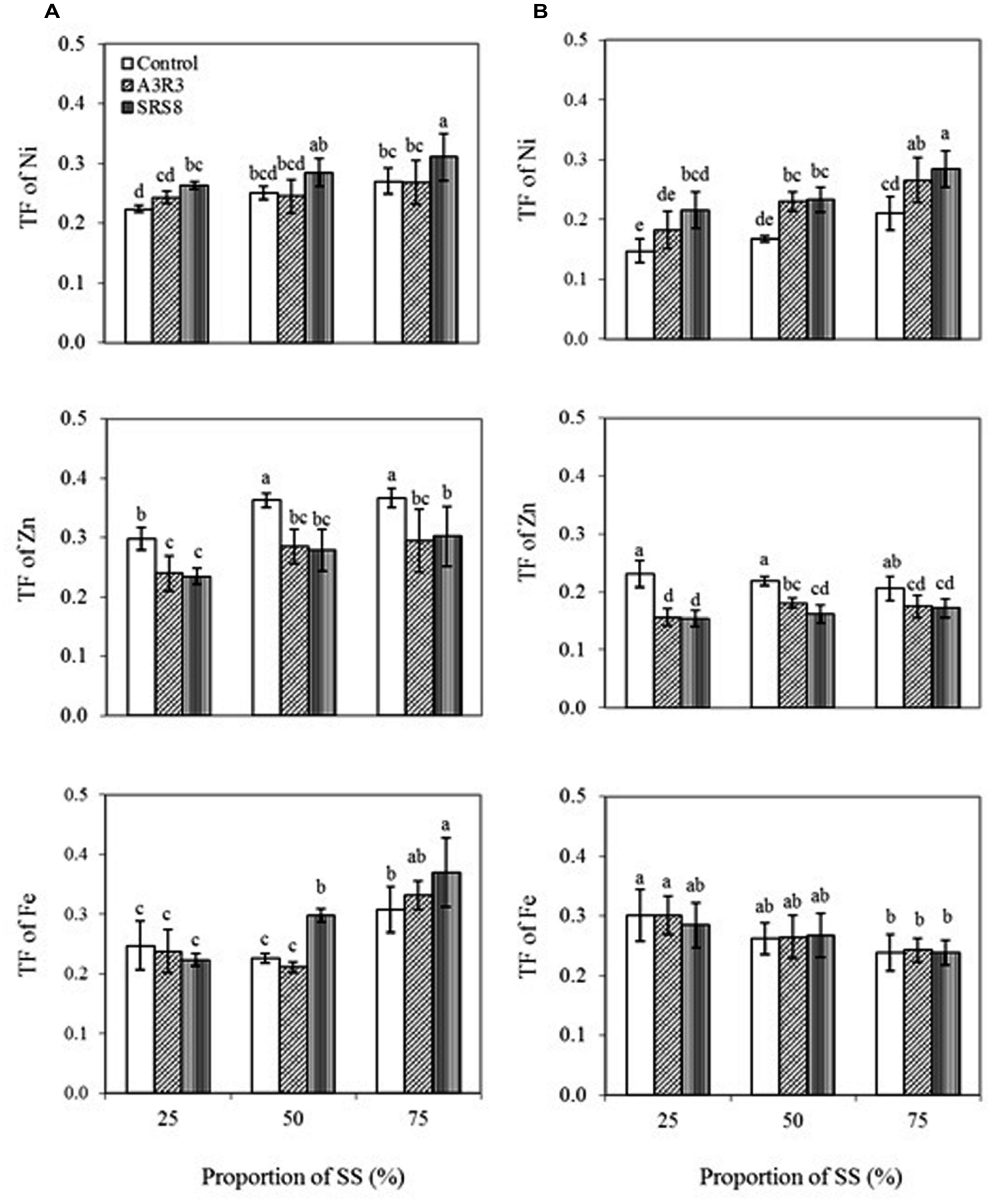
**Influence of *Psychrobacter* sp. SRS8 and *Pseudomonas* sp. A3R3 on the translocation factor (TF) of Ni, Zn, and Fe in *B. juncea* (A) and *R. communis* (B).** Each value is the mean of triplicates. Error bars represent SD. Data of columns indexed by the same letter are not significantly different according to Fisher’s protected LSD test (*p* < 0.05).

TF was calculated to assess the efficiency of PGPB inoculation on the translocation of heavy metals from roots to shoots. Plant species with a TF > 1 are considered appropriate for phytoextraction. The results showed that *B. juncea* transported relatively more Ni to the shoots than *R. communis* (**Figure 2**). This indicates that the transfer ability of Ni in *B*. *juncea* is better than that in *R. communis*, so the *B juncea* has higher potential for phytoextraction. Besides, a slight increase in TF of Ni in both plants was observed with the increasing proportions of SS. Moreover, the inoculation of PGPB strains also increased the TF of Ni in both plants grown in soils with various proportions of SS indicating that the inoculated PGPB played an important role on Ni accumulation in plant shoots. Although both *Pseudomonas* sp. A3R3 and *Psychrobacter* sp. SRS8 led to higher values of the TF, the later was more effective than the former in stimulating the translocation of Ni from roots to shoots. A recent study by Prapagdee et al. (2013) reported that the inoculation of metal mobilizing rhizobacteria *Micrococcus* sp. MU1 and *Klebsiella* sp. BAM1 increased Cd uptake into the shoot by 25 and 21%, respectively, compared with non-inoculated plants during the fourth week of plantation and indicated that heavy metal uptake/accumulation in plants strongly depended on the heavy metal mobilization potential of rhizobacteria.

In contrast to Ni, it is apparent that inoculation of PGPB reduced Zn TF in both plants (**Figures 2A,B**). For instance, the strain *Pseudomonas* sp. A3R3 reduced the TF of Zn in *B. juncea* and *R. communis* by 22 and 14%, respectively, at 75% SS, compared to the corresponding non-inoculated controls (**Figures 2A,B**). This result is in agreement with a previous report describing decreased translocation of Zn in *Nicotiana tabacum* inoculated with *Sanguibacter* sp. S_d2 grown in Cd- and Zn-enriched soil (Mastretta et al., 2009). Similarly, Rajkumar et al. (2013a) found that inoculation of *B. juncea*, *Luffa aegyptiaca*, and *Sorghum halepense* with the PGPR *Bacillus megaterium* SR28C had no effect on or reduced Ni accumulation in the shoot tissues when compared with those non-inoculated control. They attributed this effect to a direct dilution of metal concentrations by an increased shoot biomass. In our study, the significant decrease in TF of Zn caused by PGPB inoculation is probably due to the dilution effect since both plants grown with PGPB inoculation had much larger shoot biomass than those grown without PGPB inoculation. However, in case of Fe TF, the bacterial inoculation posed different effects on plants (**Figures 2A,B**). For instance, compared with non-inoculated control, inoculation of *Pseudomonas* sp. A3R3 and *Psychrobacter* sp. SRS8 increased the TF of Fe in *B. juncea* by 6 and 19%, respectively, when plants were grown in 75% SS (**Figure 2A**). On the contrary, in *R. communis*, both strains had no significant effects on the TF of Fe (**Figure 2B**). Similar results were reported by Li and Ramakrishna (2011), where the metal mobilizing PGPB *Pseudomonas* sp. TLC 6-6.5-4 was more effective on enhancing the heavy metal accumulation in maize than in sunflower. It is likely that in the present study the decrease or variation in the accumulation of Zn and Fe by the inoculated plants was not only due to the effect of heavy metals dilution but may have also been associated with other factors, including microbial populations and their response to environmental conditions in the rhizosphere, plant, and soil type, since the plants growing in metal polluted soils may modulate their growth and metal accumulation response to various physico-chemico-biological properties of the environment (Ma et al., 2011a; Becerra-Castro et al., 2012; Rajkumar et al., 2013b; Sessitsch et al., 2013; Cabello-Conejo et al., 2014). For instance, Jiang et al. (2008) recently reported that different plants, i.e., Indian mustard, maize and tomato inoculated with metal mobilizing PGPB vary in their ability to accumulate/translocate heavy metals, and found that tomato accumulated more heavy metals than other plant species from the soil. Similarly, Israr et al. (2011) reported that single metal uptake by *Sesbania drummondii* was affected by the presence of other metals. The authors attributed this result to competition between the metals for the plant uptake sites. Further studies, however, as to why these plants with PGPB have different abilities on the translocation of Fe are needed.

### BIOCONCENTRATION OF METALS

**FIGURE 3:**
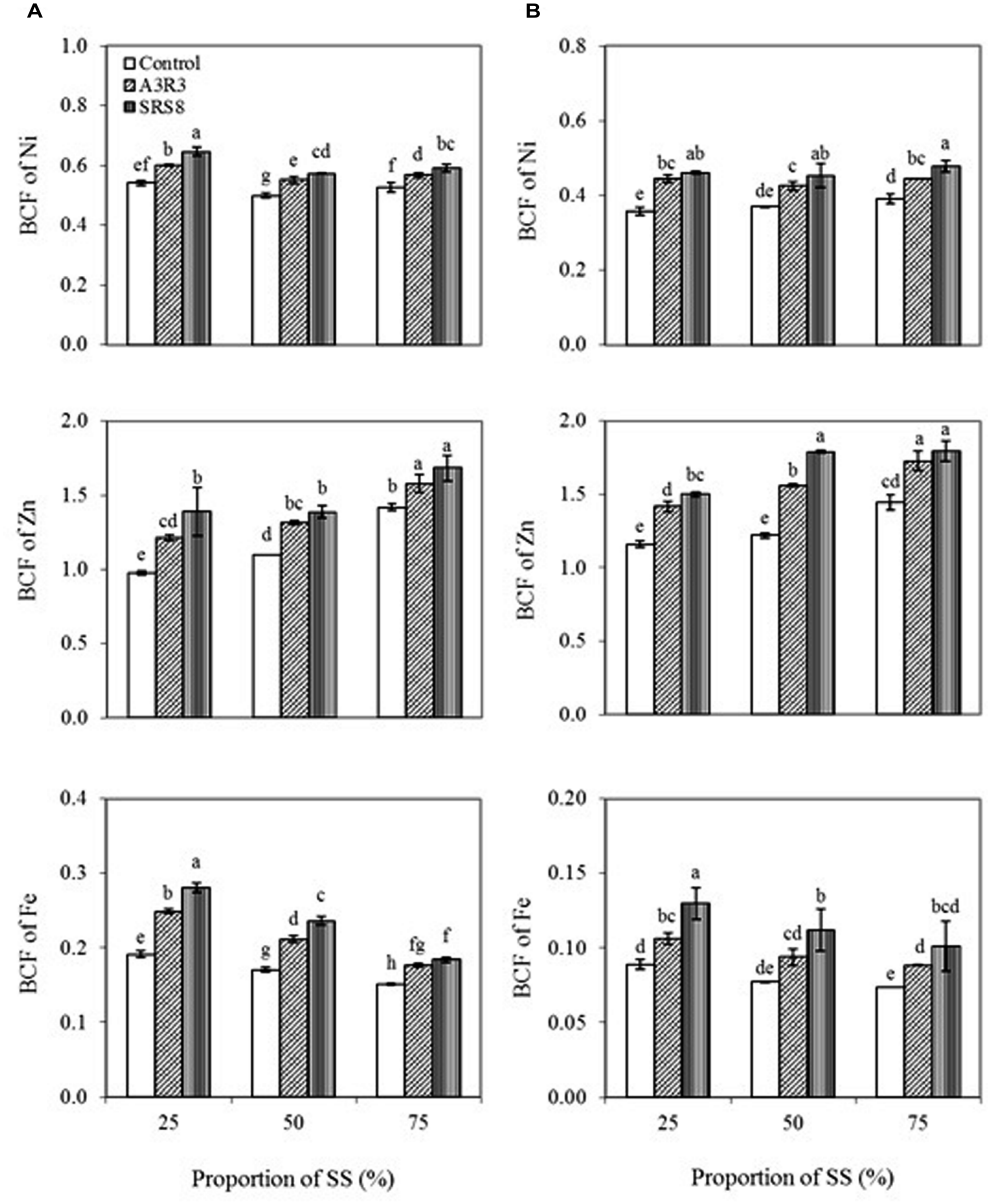
**Influence of *Psychrobacter* sp. SRS8 and *Pseudomonas* sp. A3R3 on the bioconcentration factor (BCF) of Ni, Zn, and Fe in *B. juncea* (A) and *R. communis* (B).** Each value is the mean of triplicates. Error bars represent SD. Data of columns indexed by the same letter are not significantly different according to Fisher’s protected LSD test (*p* < 0.05).

The BCF is commonly used to measure the ability of a plant to remove metals from soils (Zayed et al., 1998). In this study, BCF of Ni, Zn, and Fe in both plants increased with increasing proportion of SS (**Figure 3**), indicating that concentrations of heavy metals in soils have significant effects on the concentrations in plant tissues. Besides, inoculation of plants with *Pseudomonas* sp. A3R3 or *Psychrobacter* sp. SRS8 significantly increased the BCF of the metals in both *B. juncea* and *R. communis* compared with respective non-inoculated control. However, *Psychrobacter* sp. SRS8 was more effective on increasing the BCF of Ni, Zn, and Fe in both plants than *Pseudomonas* sp. A3R3. Based on the BCF values, overall Zn was the metal most accumulated in both plants, followed by Ni, with accumulation of Fe being very low. The increased BCF value caused by PGPB inoculation could indicate that both *Pseudomonas* sp. A3R3 and *Psychrobacter* sp. SRS8 played a significant role on heavy metal uptake and accumulation in plants. Actually, the bioavailable fractions of Ni, Zn, and Fe in SS soils where the inoculated plants grew was significantly higher than that found in the respective soils from non-inoculated plants (**Table 3**), suggesting that the PGPB increased metal solubilization also leading to a subsequent increase in plant metal uptake and translocation.

In general, plants exhibiting TF and BCF values less than one are suitable for heavy metal phytostabilization programs (Shi et al., 2011; Wu et al., 2011) because low values indicate that a given species is unable to extract large amounts of metal from the soil and translocate it to the shoots. In our study, though PGPB inoculation were capable of stimulating heavy metal accumulation in plants tissues to some extent, both *B. juncea* and *R. communis* had lower values of TF and BCF (<1) for all the three metals, indicating that these plant species with serpentine PGPB inoculation could be used for the revegetation or phytostabilization purposes.

## CONCLUSION

Although the phytoremediation of metal polluted soils is becoming a feasible alternative to physicochemical clean up technologies, it is still a challenging task because of limited plant growth and phytotoxic metal levels in soils (Kavamura and Esposito, 2010). Our study demonstrates that *B. juncea* and *R. communis* inoculated with serpentine PGPB can be potentially used for revegetation and phytostabilization of metal polluted field soils. Microcosm experiments demonstrated that inoculation of *Pseudomonas* sp. A3R3 or *Psychrobacter* sp. SRS8 improved plant biomass production and heavy metal accumulation. The former had a better performance on plant biomass production, whereas the latter on heavy metal accumulation in plants. However, both *B. juncea* and *R. communis* exhibited lower values of TF and BCF (<1) for Ni and Fe, regardless of inoculation of PGPB and proportion of SS. Overall, the increased growth response of plants in SS caused by PGPB indicates that the inoculation of *Pseudomonas* sp. A3R3 or *Psychrobacter* sp. SRS8 alleviated the toxicity of heavy metals and provided a better environment for plant growth probably through producing various plant growth beneficial metabolites. Such serpentine isolates with various PGP traits are therefore good inoculants for enhancing the phytostabilization process in metal polluted field soils. Further research will be carried out to determine the co-inoculation effects of *Pseudomonas* sp. A3R3 and *Psychrobacter* sp. SRS8 on the plant growth and heavy metal phytoremediation in degraded ecosystems.

## Conflict of Interest Statement

The authors declare that the research was conducted in the absence of any commercial or financial relationships that could be construed as a potential conflict of interest.
